# Out of Chaos—Meaning Arises: The Lived Experience of Re-Habituating the Habitual Body When Suffering From Burnout

**DOI:** 10.1177/1049732320914584

**Published:** 2020-05-04

**Authors:** Karin Mohn Engebretsen, Wenche Schrøder Bjorbækmo

**Affiliations:** 1 University of Oslo, Oslo, Norway; 2 Oslo Metropolitan University, Oslo, Norway

**Keywords:** embodiment, lived experience, phenomenology, burnout, distress, rehabilitation, qualitative, Scandinavia, hermeneutic

## Abstract

Sufferers from burnout might experience a sincere bonding to their lost lifeworld, which can result in their holding on to their previous worlds while simultaneously trying to unleash themselves. In this article, four experiential dimensions are presented in discussion with the phenomenological insights provided by Merleau-Ponty. These dimensions are “Trapped in the present body,” “the balancing act,” “precious moments of joy,” and “this is my Lifeworld now.” In the rehabilitation process, the participants demonstrated deliberate choices and reflective self-cultivation to adjust to their present situation. The illness seemed to promote a search for meaning—and out of the existential chaos, a “new” habitual body might appear. The study provides invaluable information about the rehabilitation process and the need for humanistic interventions.

I feel helpless because I’m not in control of my life. There seems to be some kind of lid on my existence. The tiredness exists in every cell in my body—all parameters are somehow on minimum. I just have to be me, lie down and disconnect from the rest of the world. It was an amazing job and fantastic colleagues. That gave me energy before, but I’m not there. The job definitely gives me a kick, but at the same time, it wears me out completely. I lie there 4-5 days later thinking about the positive things and cannot even plan what to have for dinner. There is this despair at not being able to do it. It feels like the nerve fibres don’t have any isolation anymore. Tears just stream. That’s a hard one, because the job is my whole life as I’ve lived it and want to continue living it. The realization of not being able to anymore, makes me horribly afraid. Will this empty space always be here—that darkness? Will it never pass?

Merleau-Ponty (1945/2003) sees the habitual body as it has been lived in the past—in virtue of the habitual ways in which it now relates to the world. The habitual body is also recognized in terms of what experiences it has acquired in the past. Furthermore, he distinguishes the habitual body from the present body, which might be experienced quite differently, as when suffering from burnout. Thus, these two layers of the body become the temporal point where the past, the present, and the future meet. In this moment, the past is carried forward into the outline of the future and living this bodily momentum is the experience of living the present body (Langer, 1989). The participant in the excerpt above seems to be painfully aware of the discrepancy between the habitual and the present body. In recognizing what the body no longer can do, he must face the process of modifying the habitual body. Over time, his “past” experiences of the body will eventually fade, and the present body will become the “new” habitual body. Our study addresses the lived experiences of sufferers from burnout during the process of re-habituating the habitual body.

Burnout is a condition that occurs in an occupational environment (Freudenberger, 1974) and primarily affects high achievers (Chambers et al., 2016). The condition might be understood as a reaction to long-term, job-related unresolvable stress leading to physical and emotional exhaustion, which affects the sufferers’ well-being and quality of life negatively. Research has shown that burnout can predict severe health implications, such as myocardial infarction (Apples & Mulders, 1989; Toker et al., 2005), coronary heart disease (Pedersen & Middel, 2001; Toker et al., 2005), type two diabetes (Melamedm et al., 2006), and musculoskeletal disorders (Armon et al., 2010). Therefore, it is important to pay attention to symptoms of burnout at an early stage to avoid the condition becoming chronic.

Social psychology approaches to burnout have mainly focused on the effects of different interventions (Karlson et al., 2010; Norlund et al., 2011; Stenlund et al., 2009). These approaches have shown that different factors relating to the workplace and psychosocial working conditions might have an impact on recovery (Blank et al., 2008; Karlson et al., 2014; Mengshoel et al., 2019). Few studies are conducted, however, that explore the factors associated with return to work in burnout, and the association between these factors is not made clear (Karkkainen et al., 2017). Most of the research in this field is rooted in a positivist paradigm where inherent ontological values are mirrored in how research is conducted and how data is assessed (Kelly et al., 2015). The ontological assumptions in this paradigm motivate statistical and experimental designs and causal evidence is ranked according to the reductionist presumption that any medically relevant relations go in the direction from biological processes (causes) to mental and emotional processes, behavior, and culture (effects). Although these research designs have contributed to new knowledge about how to prevent and alleviate burnout, they tend to miss much of the complexity, ambiguity, and ambivalence of individual lived experience. Moreover, most of these studies reflect a mind-body dualist split where either body or mind is always subordinate to the other (Anjum, 2016; Maeland et al., 2012; Meide et al., 2018). Further to Meide et al. (2018), psychological interventions often suggest that the self can ignore the body through a change in attitude. Similarly, in the treatment of burnout, the focus is on processes that merely take place in the mind without paying attention to the bodily symptoms (Engebretsen & Bjorbækmo, 2019). Thus, this approach does not do justice to the lived reality of the individuals who suffer from burnout.

To fully understand the experiential dimension of the rehabilitation process, it is necessary to go beyond statistical numbers and study the lived experience of the sufferers themselves. In a dispositionalist framework, conclusions about individual propensities related to treatment cannot be inferred directly from a statistical distribution. This means that unique individuals cannot ever be a statistical average (Anjum & Mumford, 2010). To infer causal connections within a rehabilitation process, the causal mechanisms must be understood in relation to the whole person in his or her environment as both individual and relational, as well as contextual.

There are few qualitative studies regarding the experiential dimension of the return to work process among individuals suffering from fatigue- and pain-related diseases in general (Norlund et al., 2011). We will refer to some of them later in the text. A synthesis of qualitative research findings shows that living with chronic illness can be understood as an ongoing, continually shifting process (Paterson, 2001). This suggests that the experiences of burnout change over time. Although some of the individuals who suffer from subjective health complaints such as burnout do recover and manage to get back to work within the sick-leave period (52 weeks), research has shown that the recovery process is often demanding and time consuming (Mengshoel et al., 2019; Øyeflaten et al., 2012). Only a few studies have explored how the sufferers from burnout deal with their condition. To contribute to fill this gap, the aim is to explore how sufferers from burnout who have been on long-term sick leave (>52 weeks) deal with the process of coming to terms with their present body—the lived body as ill. In contrast to mainstream research on burnout, this study will emphasize the context within which individuals who experience burnout live and how they make meaning of their situation. We argue here that their lived experiences will give invaluable information about the rehabilitation process.

## Method

### Study Design

In this work, we are inspired by the phenomenology of Merleau-Ponty. He stressed the need to understand the person as a body-subject, with consciousness always embedded in the body (Merleau-Ponty, 1945/2003). This paradigm assumes that reality is not objective but subjective and that individuals create meaning from their lived experiences. Phenomenology as method is consistent with the constructivist research paradigm in accepting that the subjective processes of human experience provide a source of explanation of human action. In addition, personal descriptions of experiences and opinions are a legitimate source of research data (Creswell, 1998).

Both Merleau-Ponty’s and van Manen’s ideas form a theoretical basis for much contemporary work in phenomenological psychology (van Manen, 1990). Phenomenology primarily aims to describe the lived world of everyday experiences (Finlay, 2011) and to “show” how meaning reveals itself (van Manen, 2014). This method employs a naturalistic inquiry that attempts to inductively and holistically understand human experience in context-specific settings (Patton, 1990), generating data rich in detail and embedded in the context (Creswell, 1998). These philosophical theories do not just inform the phenomenological methods: the findings that these methods generate can be analyzed using these concepts heuristically. Further to van Manen (1990), theme analysis is an act of insightfully “seeing” meaning, rather than being a rule-bound process. Thus, by applying a phenomenological method, a deeper analysis of the data and a move from description to interpretation is enabled (Langdridge, 2007).

### Recruitment Procedure and Interviewees

The first author contacted 10 psychotherapists from the Oslo area (Norway) and asked if they could help to recruit participants for the project. They were given written information about the project and a handout for their clients with contact information. Eight volunteers, two men and six women, made contact. They were invited to a personal information meeting. The aim of this meeting was to present the project and make sure that they fulfilled the selection criteria. These criteria selected for individuals between 25 and 65 years who had been employed at least at an 80% position and had been on sick leave for more than 52 weeks prior to the interview. In addition, their burnout symptoms should be consistent with Exhaustion Disorder according to ICD-10, F43.8A (World Health Organization, 2000). All the volunteers fulfilled the selection criteria and agreed to participate in the study. In addition, they were invited to keep a personal diary during the fortnight prior to the interview, which they were free to refer to during the interview.

### Data Generation and Analysis

The focus of the study was to explore the participants’ lived experience of struggling to re-habituate the habitual body and to come to terms with their lived body as ill. Semi-structured interviews were chosen to uncover how the participants made meaning out of their situation. The research question was “how do you experience being yourself in the rehabilitation process related to encountering others—and what factors enhance or restrict quality of life?” This question was followed up during the interviews, which lasted between 90 and 180 minutes. During the interviews, an important consideration was to meet the participants in reciprocal humanity, while being aware that the interaction would influence the dialogue. Due to their illness, they were given the opportunity to choose the venue for the interview. Four interviews were conducted at the first author’s office and the other four at the participants’ homes. The first author conducted the interviews and transcribed the audio-recorded interviews verbatim directly after the interviews. The participants were issued with a copy of their transcript. They had the opportunity to not only read but also amend the transcript if they wanted to do so for whatever reason. Two of the participants used this opportunity to make parts of their lived experiences more explicit.

We applied a phenomenological research method, which emphasizes the individual lifeworld (Finlay, 2011; van Manen, 2014) and the lived experience descriptions provided the lifeworld material for the phenomenological inquiry. Hermeneutic reflection involved practicing heuristic activities as outlined by van Manen (1990). The process of phenomenological reflection and analysis occurred primarily in the attitude of the *epoché*, the reduction and reflection on the meaning of lifeworld experiences (van Manen, 2014). This process in turn led to the creation of experiential themes. Within the attitude of epoché, the aim is to keep a stance of openness toward the data—to set aside or “bracket out” personal presuppositions. The experiential themes bring attention to or exemplify the nature of the experience. This method is especially relevant when the aim is to explore rehabilitation as a lived phenomenon. During the interviews and throughout the data analysis, the intention was to allow any emotions, patterns, and themes related to the interviewees’ experience to emerge, rather than being based on predefined themes. In this process, the aim was to open up for meaning, explore, and elaborate on the participants’ pre-reflective lived experience. In the first step when analyzing the data, the focus was to immerse ourselves in the participants’ lived experiences, while listening, reading, and reflecting on the meaning of what the participants shared in the interviews. During the second step, thoughts, comments, and questions were highlighted in the text to surface our preunderstanding and potential prejudices. As the researcher is part of the field, it is challenging to be able to set aside one’s own presumptions of how this phenomenon could be understood. Therefore, when analyzing the data, the intention was to keep a stance of openness toward the participants’ expressions of their experiences. Thus, the process of analysis and interpretation as a non-linear style was concerned with the dynamic relationship between the part and the whole of the empirical material at multiple levels (Finlay, 2011).

The data analysis might be understood as a process of insightful invention to uncover meaning. As part of this process in step three, key phrases and meaning units were identified and clustered into phenomenological themes. These themes were developed as a description of an aspect of the experiences that were found in each cluster of meaning units and contained a moment of the experience. Thus, these themes can be understood as the meaning structures of the experience, which formed the basis for the thematic reflection.

Reflective writing was part of the fourth step, where the phenomenological themes were used to compose textual descriptions. In phenomenological writing, lifeworld experience descriptions are re-written and tightened to show, rather than tell the meaning of the experience. Van Manen (2014) refers to this crafting process as writing anecdotes, which intends to speak to our imagination and give us a flavor of the phenomenon. Further to van Manen (1990), a phenomenological text is ultimately successful only to the extent that we, its readers, feel addressed by it. Hence, the text must reverberate with our ordinary experience of life as well as with our sense of life’s meaning. Although human experiences are always more complex than what is captured by writing alone, the text as a whole is intended to represent the findings of the phenomenological exploration (van Manen, 1990).

### Consent and Other Considerations

The project was ethically approved by the Norwegian Centre for Research (NSD no. p469). Prior to the interviews both verbal and written information about the study were given to the participants in separate meetings, and written informed consent forms were signed by all of them. Clear boundaries were set about the context of the research project, the research process, and how the findings would be reported. According to the standards set out by the Norwegian Centre for Research and the University of Oslo (UiO), all data were de-identified and stored in TSD (Services for Sensitive Data). In designing the study, the participants’ vulnerable situation, being on long-term sick leave, was given special consideration. We acknowledged that the research process could cause painful awareness of their situation, and consequently, the participants were informed about the opportunity to come in for a debriefing session after the interview if they experienced excessive emotions as a response to what was brought up during the interview. However, this situation did not occur.

The following section provides narratives of the participants’ lived experience of struggling to re-habituate the habitual body and to come to terms with their lived body as ill.

## The Process of Re-Habituating the Habitual Body

As the participant states in the introductory section, his job is his whole life and how he bonds with his work seems quite sincere. This is more or less in line with what the other participants described in the interviews as well. All the participants have experienced symptoms of burnout for several years and all except for two were on work assessment allowance when the interviews were conducted. During the sick leave, all of them have felt being pushed to go back to work, either full-time or part-time, despite not feeling well. What we have seen in the participants’ narratives, the backdrop of having ended up with a fatigue reaction, is their dedication to work. We will here give a detailed description of the participants’ lived experiences of the rehabilitation process. During the phenomenological analysis, textual descriptions containing the meaning structure of the experiences were composed. These meaning structures are presented in discussion with phenomenological insights provided by Merleau-Ponty (1945/2003) to highlight how meaning can be understood phenomenologically in relation to the rehabilitation process. As our intention mainly was to highlight meaning, the names of the participants are left out. The textual descriptions are presented in italics and the phenomenological insights in plain text.

### Trapped in the Present Body

The healthy body can be described as transparent, as we have a pre-reflective sense of certainty that our body will support us in the activities that we engage in. In the usual course of events, the functionality of the body is taken for granted and not attended to. When experiencing illness, however, what the body is capable of doing is altered. Everything feels changed; the body does not only feel different now, but also how it has been lived, what it has been capable of, and how it has been experienced in the past, have changed. *That awful feeling of being trapped started five years ago; the panic I feel when I have to do something but have no energy. I know what I have to do, but I cannot manage it. I can give it everything, but still it isn’t enough.* When “I cannot” is frequently confirmed, the certainty of “I can” might change into “I might be able to” due to the awareness of the experienced inability. When being unable to act as before, the focus of our attention becomes on what we are not able to engage in. Recognizing the body becomes a deliberate focal point of attention. When becoming consciously aware of what the body no longer enables us to do, the person must modify his or her actions. *My head gets tired quickly and I get fever after the slightest effort. I actually got worse throughout the autumn when I worked 25%. That didn’t work out at all. So now, I’ve been told by both doctor and psychologist to learn how to do nothing.*

The body can no longer be taken for granted or ignored. It must explicitly be attended to in various ways. The body completely determines the daily activities. This experience might result in a sensed narrowing of the felt body. *When the “Bang” feeling strikes me, I feel trapped. The energy disappears and I am aware that I become irritated and impatient because I realize I cannot do anything about it. I just have to get through it as best I can. I have lost courage somehow. I don’t know how long I can stand feeling like this.* Illness triggers a fundamental change in the relation between self and body. A person who suffers from burnout might be especially sensitive to signals of fatigue. In illness, the body appears to be out of control of the self and seems to demonstrate an opposing will on its own. *This process is very hard. All the time I have to be strict with myself and pull myself together. My whole body feels like a wrung-out mop. I am absolutely wiped. It’s as if you flip a switch and all the energy is gone. It’s completely unpredictable.* The bodily unpredictability makes it impossible to implement time for working. Therefore, the person who suffers from burnout must hand himself of herself over to the unpredictable body. In the experience of illness, the significance of past, present, and future is changed, which is experienced as a chaotic disturbance in the person’s world. Individuals who suffer from burnout might talk about their body as being separated from the self. The sufferer seems to split the body to re-connect with the body. *At this moment it’s no fun. I’d rather get away from being me, as I’m neither the work person nor the private person. I cannot manage anything anywhere. I can’t accomplish what I want to do. It’s shitty to be half a man to put it like that. That’s heavy.*

Illness is experienced as a disruption of the lived body. The disruption causes the sufferer to explicitly attend to his body as body, rather than simply living it unreflectively. The body is thus transformed from lived body to object body. When no longer being simply lived unreflectively, the body is suddenly perceived as a thing that is external to the self. This objectification might be experienced as an alienation of the lived body. This disruption of the lived body strikes at the very self. *I can’t get out of it—I am stuck . . . Not being good enough.* The felt change in how the body is experienced can be difficult to accept because the body is the medium through which we interact within the world and express ourselves. Illness generates feelings of helplessness and represents a concrete loss of independence. This change is profoundly felt, not only as a loss of bodily integrity but also more importantly as a diminishment of selfhood. *I spend a lot of time on this sofa and that worries me. How do I get out of it? I feel stuck here—as if my world is this sofa. I am in a situation where I cannot go out into the world on my own two feet and take part. I become invisible, because I’m not joining in. What I really want is to be outside, be active and moving around, and to be with others.*

### The Balancing Act

Phenomenology accepts the human being as an agent existing in continuing interplay within the “organism-environment-field.” The organism-environment-field is defined as a systematic web of relationships, which may be understood as a totality of mutually influencing forces that together form a unified interactive whole. Thus, the human being is in constant interaction with his or her environment, aware of the immediate-present phenomena such as the experience of bodily sensations in response to internal and external influences. *I have felt very resistant and been reluctant to sit down. On a day where I feel I’ve got a bit to give, I can sit down and see what happens. I’m glad I have had those pieces of work lying there and had the opportunity to do one page and then stop—that was today’s task.*

Whereas our embodied capacities ordinarily provide the background to the figure of our worldly involvements, in illness the body becomes itself the figure of our intention against which all else is merely background. In addition, the primary meaning provided by the body may be disrupted. A person who suffers from burnout must relearn to become aware of his or her bodily responses by either monitoring or sensing the body. Monitoring refers to a deliberate activity while sensing is more passively becoming aware of physical sensations. Through the bodily signals, they can experience that they are their body. *I am very good at enduring, but I do become very exhausted. I’m not aware of it there and then. It can hit me tomorrow—or the day after. The problem is that I don’t know my own limitations. Now I try to be more aware; “I’m going to do this,” but I have to spend three days doing it. Not do it in one morning.* The body in the words of Merleau-Ponty is “an expressive unity, which we can learn to know only by actively taking it up” (Merleau-Ponty, 1945/2003, p. 239). Thus, bodily alertness implies that reflection is involved in almost everything we do. Even ordinary tasks require attention because of overwhelming sensory perception that might cause overload and result in a fatigue reaction. *I have been forced to set boundaries and say “no, just can’t do this.” When I start getting a little energy back, I go all out, but then I start to check with myself and decide OK—I’ve rushed it. So, in fact I have to force myself to think “let’s do only this. And leave the other stuff until tomorrow. Or, another day.”*

The primary perceptual relation between the body and object is that of form giving. Sensory perception is already charged with meaning in that the object is always grasped as a significant whole against a background of co-perceived things. Any change in movement results in a change in the background attitude. The field is dynamically changing, and the person is constantly engaged in contact processes to adapt to those changes. *It feels very good to go out and putter around with one thing or another. All the time I keep in mind how setting fire to all the gunpowder will not bring me forward. I think about this almost every day. Am I doing too much now, or not? This is where I do my balancing act*. The notion of purposiveness and intentionality is essential to embodiment. Therefore, the lived body exhibits an if-then temporality of bodily action to predict contingency. When healthy, we act in the present in light of more or less specific goals, which relate to future possibilities. In illness the character of lived temporality changes and bodily intentionality is thwarted when the “I can” is experienced as “I cannot.” Everything that affects my body affects me and becomes bodily if-then experiences. Because the taken-for-granted, if-then causality of the body is interrupted, the purposiveness is also disrupted. This interruption results in a focus on the present moment and we become unable to effectively project into the future.

### Precious Moments of Joy

The cognitive capacity of the lived body should be understood in terms of a bodily knowledge that allows us to perform everyday activities and engage with others in meaningful ways. Through its intentionality, the lived body grasps and relates to the world as a world of meaning. *I have to make the best of what my life is right now. And, my life at the moment means that I am able to do very little. Last Sunday I spent some time sitting comfortably in the sunny winter weather and watching my children play in the garden. I was served “cream-cakes.” Taking part in their play was really nice and I want to join in more often*. When suffering from burnout the body is experienced as a sensitive source of information that alerts us to slow down. In some situations, however, the bodily awareness can be put on hold, which gives openings for bodily enjoyment. *Despite it being terribly tiring, I decide to go to a café and to do things I enjoy. Even if I have to go home again straight away, I have tried. I don’t allow myself to give in. To get going and do the little that I can manage is an important motivational factor.* The unpredictability of the body demands that we plan all daily events. Even joyful moments require conscious planning. The person suffering from burnout may refuse to be “dictated to” by his or her body. Despite the limits imposed upon them by their body, freedom is enacted which brings moments of joy into life. *As when the whole family stay together in our cottage, I rest while they are skiing. Afterwards when we enjoy meals together life feels good. I long for the day when I will feel healthy.* The lived body is just experienced as a limitation when you are unable to do what you want to do. Thus, well-being might temporarily be experienced when the body functions as before.

When we become aware of and respond openly to our embodied relational existence, we tend to perceive the animal world as a shared community. This field is inhabited by kindred others, who are calling for our conscious attention and ultimately for our love. *The interaction with the dog—the fact that someone is depending on me and loves me unconditionally makes me want to pull myself together and take her out to train a bit. I just enjoy what we are doing. That makes me happy.* The invisibility of burnout can create a need in the sufferers to justify their condition to others. This need may be enforced by the fluctuations in symptoms, as they understand that their condition might be confusing to others. *I think it’s very odd that I can cope with the dog because I can’t really tackle anything else. I don’t know where I would have been without it, because then I wouldn’t have had a sense of belonging or achievement anywhere. I am very grateful and happy for having the dog to hold onto. I think it has been an important factor for keeping me going and not giving up.*

Language as both spoken and bodily expressed is destined to play a crucial role in the perception of others. In the experience of dialogue, a common ground is created between the other person and me. My thoughts and his/hers are interwoven into a single fabric. My words and those of the other are inserted into a shared operation and becomes an emergent phenomenon. *I feel that I have had a long, hard road to walk and I have walked all this way alone. I’m getting closer to a light in the tunnel. That’s because I have been able to share what I have in the baggage with the psychologist and my husband.* In the dialogue with the other, we become a dual being, where the other and I are no longer separate. We are collaborators for each other in a divine reciprocity. Our perspectives merge into each other and we co-exist through a common world:

In the present dialogue I am freed from myself; for the other person’s thoughts are certainly his; they are not of my making, though I do grasp them the moment they come into being, or even anticipate them. And indeed, the objection which my interlocutor raises to what I say draws from me thoughts which I had no idea that I possessed, so that at the same time that I lend him thoughts he reciprocates by making me think too. (Merleau-Ponty, 1945/2003, p. 413)

Being able to open up for co-existing through a common world can be experienced as a healing effect. I did not see light anywhere and needed someone to see me and help me. That November day at the psychologist’s when she suggested sick leave, at last there was someone who held me—who carried me. There was this need in me for that to happen. And I have managed to open up and tell my husband what I need instead of telling him how inept he is. I have been terribly angry with him for a long time, but now something is happening. When he supports me, I can sense the healing effect.

The perception of other people and the intersubjective world is problematic only for adults. The child lives in a world, which he or she believes accessible for everyone. They have no awareness of themselves or of others as private subjectivities. They subject their thoughts to critique without attempting to criticize the other. For them a self-evident world exists where everything, even dreams, take place. *It’s only in the last two years that I have managed to focus more on myself again and to look after myself. That’s when we chose to foster a young boy. He’s been an absolute marvel, a therapist de-luxe who has helped me get out of the black hole. This little man, who’s all shiny and new, can take me to a whole new place. And then the world turns a little bit and starts to give some sort of meaning.* In search of meaning, we are ourselves the meaning-maker of the field.

### This Is My Lifeworld Now

In illness, the sufferer from burnout comes face-to-face with the radical contingency of his or her existence and the inescapable limitations of their embodiment. *When you have so little strength, it becomes easy to think that others should help you. Like you are looking for something from outside. And they are not there. Nobody except myself can make me feel better.* Freedom to act is situated in such a way that there is no freedom without a field—and since we as embodied subjects are of the world with which we are in constitutive relation, it is not outside ourselves that we are able to find a limit to our freedom. *But how far can I manage to heal myself or to accept myself? I don’t want to be such a weak and incurable person. I would rather be strong. So, it means having to acknowledge a chapter that is finished. Which is part of accepting and carrying on. It’s hard, but how long should I go and grieve for that job and that I drove myself to fall flat on my face? Some time I just have to say stop*. Whether we want it or not we must learn to accept and deal with the physical limitations, which our illness imposes upon us. *It is so hard to accept that what has happened has actually happened, since nobody else accepts it. I ponder long and hard over how I will be able to come to terms with it. I am still optimistic; thinking that over time things will sort themselves out. Even if I don’t exactly see that now.* In a critical way, we are forced to recognize our inherent vulnerability. The sense of inescapability and limitation is intrinsic to illness-as-lived. *There is no guarantee there will be a cure, so until I know it will have to be as it is. I can’t do anything about it. Right now, this is my life.*

Phenomenology provides, through accounts of how we are born into a world already inhabited, shaped, and made meaningful by others, a way of understanding how human existence is characterized by basic openness to others and the world. *I do go around pondering a bit about stuff in my everyday life, and I have found that if I don’t reflect, I will stagnate. There is only one way to get out of this and that is to grab hold of it and do something about it. And by reflecting I can discover what I can do something about.* Our very perception of something as a choice needs to be understood against our whole situation including our bodily capabilities, our goals and plans, as well as our perceptions of others, the world, and ourselves. *I see that there is no space for a job right now. It hurts because it’s not where I wanted to go. It hasn’t come easy for me to accept the situation instead of working against it.* The intentionality of the lived body constitutes it as fundamentally open to new possibilities and to forming its own style of being in interconnection with its conditions. *As I’m getting a bit better, I am more aware of the consequences of what I’m doing. Eventually I learn to look after myself. I’m much more conscious of what I need and what the meaning of life really is instead of just going along for the ride. I feel more in charge of my own life.* According to Merleau-Ponty, to grasp my lived body, as body requires an act of reflection, which necessarily transforms it into an object-body, which is the experience of how my body might be perceived as an object in the gaze of the other. *For myself I hope I will become better at voicing my own opinions. But that part is pretty hard because I am worried about disappointing and to not live up to expectations.* When ill, I recognize the brute fact of my being as material stuff. This experience is one of alienation. As an object for others, it altogether escapes my subjectivity.

The situation might be experienced as radically changing and perhaps forcing the enactment of non-habituated action and movement. *I am not as ill today as I was six years ago. Today I can put my foot down and manage things better than I did then. It has become a necessary lesson, because I believe this was a reason for ending up in that burnout ditch.* Even if my bodily style of being makes certain choices and decisions more likely for me than others, there is room for creative adjustments. *At least I have learnt not to set off all the gunpowder at once. Even if the urge to be in the flow from the past is there, working 100% is impossible. But I would like to work as much as I am able to. I guess that’s what I would like back—if not life as it once was, but the feeling of managing my own life.* Merleau-Ponty’s description of the lived body and its compartment in the world and relation to others offer a rich account of bodily integration. When Merleau-Ponty insists that to be born is both to be born of the world and to be born into the world, he captures how human beings exist in a double way of being both already constituted with a certain meaning and at the same time themselves constituting meaning. *It no longer worked giving so much and receiving so little. Then the long, tiring and redemptive process of pulling the threads in the chaotic ball began, and slowly I started to understand that I was too hard on myself. There’s a lot I can’t change, but I can alter my own attitude to myself. That in fact is what is happening. At least I have a small and growing protest inside me about not being so insignificant.*

## Out of Chaos, Meaning Arises

In this study, we used a phenomenological approach informed by the insights of Merleau-Ponty (1945/2003) related to the lived body. The aim was to explore how sufferers from burnout who have been on long term sick leave (>52 weeks) deal with the process of coming to terms with their present body. We have seen how the illness obstructs the participants’ ability to live according to their assumptions about their bodily existence as well as their inability to free themselves from the facticity of suffering burnout. Thus, when unable to fulfill the desire to enact habitual actions, loss of future possibilities was experienced as a constriction to their lifeworld. The participants’ lived experiences show how the very nature of the body as being in the world is transformed, and the fundamental unity between the body and themselves is experienced as disrupted.

Carel (2012) points to illness as an intruder that alters the person’s very being in the world. Due to a loss of bodily transparency in illness, sufferers from severe diseases are forced to relearn how to balance worldly demands, as the previously experienced “I can” no longer can be taken for granted (Carel, 2013). The experience of bodily doubt constitutes the transition from bodily capacity to bodily incapacity and creates an anxiety on a cognitive level as well as on a physical level (Carel, 2013). This is in line with how Toombs (1995) has described illness in her work. In degenerative diseases such as multiple sclerosis (MS), the disruption of the lived body effects the taken for granted awareness of the social world of everyday life. Thus, the bodily disruption causes a disorganization of the person’s self and his or her lifeworld (Toombs, 1988). Other researchers have also mentioned the struggle to maintain balance between demands and resources in a fight for survival when suffering from fibromyalgia (Raheim & Haland, 2006; Søderberg et al., 1999) and burnout (Ekstedt & Fagerberg, 2005). These previous findings align with the phenomenological descriptions presented in our study.

We have also seen how the participants objectified their lived body in various ways while living through illness. They described the lived body as an object outside their subjectivity in the midst of a world that was experienced as alienated. This experience might be recognized as a state of living in-between (Ekstedt & Fagerberg, 2005; Jingrot & Rosberg, 2008). Svenaeus (2000) refers to the loss of bodily integration as the gradual loss of homelikeness. This process is recognized as one of self-alienation (Leder, 1990) as well as a feeling of a split between body and mind (Svenaeus, 2000). Søderberg et al. (1999) show in their work how the loss of bodily integrity, loss of control, and freedom to act might lead to an existential breakdown in the familiar world. This finding is supported by Jingrot and Rosberg (2008). Their study also shows that the gradual detachment from the interviewees’ lifeworld could be understood as a process of losing one’s homelikeness, which ended up in an existential breakdown. Correspondingly, the participants in another study described their experience as a fundamental collapse of their lifeworld (Lian & Lorem, 2017). Our study supports these findings as we interpret that the body image not only is an experience of the participants’ biological body, but of their lived body. Therefore, a threat to their lived body necessarily incorporates a threat to their very selves, which in turn ended up as a bodily disintegration. Thus, the disruption of the lived body is not merely experienced as a breakdown in the mechanical functioning of the biological body. As the participants’ lived experiences show, the significance of past, present, and future is changed, which seemed to be experienced as a chaotic disturbance in the participants’ world. In the words of Merleau-Ponty (1945/2003), “in illness it is the intentional arc that goes limp” (p. 136). Thus, a new lifeworld must be re-made.

As our phenomenological analysis shows, the inherent human ability to adjust to the never-ending temporal processes of life seems to take place as the participants demonstrated deliberate choices and reflective self-cultivation to adjust to their present situation. By being able to demonstrate new habitual actions, the participants might be able to constitute a “new” defined self, which in turn might result in a re-habituation of the lived body. Similarly, Svenaeus (2014) asserts that suffering from pain may be transformed when core life values are changed by a reinterpretation of the life-story, which eventually might ease the experience of the present lifeworld. Finlay and Molano-Fisher (2008) correspondingly conclude that Pat in their study had to learn to cope with her transformed self to come to terms with her past, present, and future being.

All the participants seemed to be closely tied to their previously lived worlds through their bond with their work. Although the illness had put an end to this sincere relationship, the lost world was still present in their lived bodies through intentionality. In longing for the experienced past, it seems that the intentional direction is pointing “backwards” in time instead of “forwards,” which might restrain the reorientation and ability to act. The participants struggled to free themselves from their memories of the past and seemed to vacillate between two poles: holding on to their previous worlds and unleashing themselves. Honkasalo (2000) in her study refers to the pain experienced in the past as still present in her participants’ lived experiences, showing that they hold on to their previous worlds. She refers to this experience as a refusal of mutilation, which can be understood in the light of Merleau-Ponty’s (1945/2003) description of a man with a phantom limb. Through his description of this man’s relation to the past, we get an image of the ambiguous presence of the lost. His description illuminates our understanding of the intentional body. Accordingly, this highlights how demanding the participants might experience being stuck in the past while simultaneously being forced to relate to a lifeworld that is changed.

Our phenomenological analysis also shows how the participants are reflecting on their changed life situation and how to cope with it, which enhanced their self-understanding. This in turn seemed to empower non-habituated action and movement. Even if their bodily style of being makes certain choices and decisions more likely for them than others, there seems to be room for creativity. Once we accept the existence of a subjective phenomenology, the human being must be conceived in terms of an autonomous agent, aware of his or her bodily responses in relation to different ways of taking care of himself or herself. As our study indicates, the lived body possesses its own operative intentionality of habituated actions where the interaction between the participant and his or her environment-field is a contact process. Through this process, meaning emerges. We saw that the participants managed to engage with others despite struggling to survive. This is especially evident in the participants’ experienced precious moments of joy where an awareness of well-being seems to evolve into consciousness. Thus, the participants exist within the continuum of awareness and consciousness of constantly shifting experiences, which can empower non-habituated actions. Their mode of being-with-others seems to form and constitute their lived experience positively. In search of meaning, the participants became aware that they themselves were the meaning-makers of their lives—and out of the existential chaos, new meaning appeared. This finding is similar to what Salminen et al. (2015) describe in their study where the analysis revealed the overarching theme: “my well-being in my own hands.” Their findings showed that accumulation of support led to a revival of joy in the participants’ lives, which in turn enhanced the participants’ experience of well-being and perceived control. Despite suffering from a different type of illness, long-term users of physiotherapy similarly expressed their own meaningful ways to recover (Mengshoel et al., 2019). Another study concludes that internal and external resources are intertwined and can directly increase the probability in patients suffering from exhaustion disorder to regain ability to work (Norlund et al., 2013).

## Final Comments

The findings in our study suggest that the prevailing biomedical understanding of burnout in the health care system does not adequately capture the personal experience of the syndrome, which in turn restrains the participants’ ability to integrate the bodily experience of burnout into their lives. This finding is in line with previous findings of how persons living with disabilities struggle to contextualize their disabling condition (Gibson et al., 2005) and how the disabling disease and not the lived experience is the focus of interventions and research (Lutz & Bowers, 2005). The reason for this fact can be the limited focus of the biomedical model on the heterogeneity and complexity of syndromes such as burnout.

Burnout is often labeled as “subjective health complaints” and the exact physiological causal mechanism is still unknown. The symptoms are complex with seemingly individual combinations that often persist and fluctuate in intensity over time. Due to the lack of a known biomarker in burnout, the medical system and the social security system seem to agree that the subjective health complaints are often due to psychopathology (Åsbring & Närvänen, 2003; Lian & Lorem, 2017; Maeland et al., 2012; Nettleton, 2005). Thus, working is considered part of the rehabilitation process, as the participants are not recognized as physically ill.

As the participants’ lived experiences show, the rehabilitation process is time-consuming. In Western societies, the political health economic imperative is to reduce costs. In Norway, there is a focus on shortening the rehabilitation process by focusing on treating depression, which is a common symptom in burnout (Bianchi et al., 2015). The treatment is based on psychotropic drugs (Selective Serotonin Reuptake Inhibitors) in combination with psychotherapy and graded exercise. The psychological interventions are mainly based on cognitive behavioral therapy, which aims to provide the patient with the necessary self-confidence to cope with their condition (Alderson et al., 2012). The effects of these interventions have however proved very small (Coventry et al., 2013; Smith et al., 2012). This fact can be related to how cognitive behavioral theory ascribes autonomy to the person from an outside perspective and additionally expects that the person at any time might be able to change his or her situation (Kall & Zeiler, 2014). Such expectations lead to social injustice because they can constrain autonomous choices.

Phenomenology does not refer to an autonomous self, but to situated freedom and the interplay between freedom and facticity. Sartre’s account of freedom (Sartre, 1958) emphasizes that although we may have little or no control over our illness, we always have freedom to choose how to respond to the difficulties within our lives, and how to constitute the likelihood of feeling better. In this regard, Sartre sees embodiment not only as radical limitation but also as possibility. The recognition of those aspects over which it is possible to exercise some control may provide hope. Thus, Sartre’s (1958) account of the contingent necessity of embodiment may provide an affirmative response: the body is at once, “the necessary condition for the existence of a world and the contingent realization of this condition” (p. 462). Therefore, to be able to support persons who suffer from burnout in their rehabilitation process, we need to consider their lived experience as well as how they make meaning of their situation (Engebretsen, 2018; Lutz & Bowers, 2005) as an interplay between freedom and facticity. When the caregiver acts as a human being and a supportive ground the person can experience that internal and external resources are intertwined, which in turn can facilitate necessary changes to adjust to the present situation. This approach can empower the person to live through the existential crisis and support his or her integration of the past, present, and future lifeworld. As the participants’ experiences in this study highlight, the existential breakdown can provide a starting point for rehabilitation and self-acceptance, as also presented in Aroll and Howard’s (2013) study. This finding is in line with what Sartre refers to as the contingent necessity of the body.

Although the findings in this study provide new insights into the rehabilitation process of sufferers from burnout, additional research related to what factors enhance or restrain the empowerment of the unique individual is needed. When the feeling of being restrained becomes the figure against the background that we long for, we might become aware of how the absence of interaction deeply affects us. This study has shown how some encounters profoundly affected the participants’ self-affection. Our hope is that this knowledge will contribute to provide a deeper understanding of human needs. Taking the lived experiences of sufferers from burnout into consideration can prevent symptoms from becoming chronic, which in turn will improve the prognosis for recovery. Regarding further research on burnout, progress calls for a deeper exploration of how inter-affectivity can influence our affective intentionality. This includes a focus on how the caregiver can contribute to such transformative changes and how this knowledge can be incorporated as part of a rehabilitation program.

## Methodological Considerations

In this study, we aimed to represent the participants’ experience of their rehabilitation process as closely as possible to how they themselves made meaning of the situation. We recognize that the overlapping role of being a researcher, interviewer, analyzer of data, and author of the resulting narrative purposely becomes a vital part of the study, which however can become a threat to validity. Another factor to take into consideration related to this study is linked to the recruitment procedure. All the participants except for one had been in psychotherapy during their illness. This fact could have resulted in raised awareness of their lived experience compared to a less heterogenic sample of interviewees. In addition, this raised reflexivity of their situation could have contributed to their willingness to participate in the research project to tell their story.

Researcher bias and the effect of the researcher on the participants, also referred to as reflexivity, are mentioned as two main types of threats to validity (Maxwell, 2013). Therefore, in this study, we paid attention to the need for increased awareness of integrity and accountability regarding the research relational process. Consequently, in this study, we have tried to demonstrate trustworthiness—a quality that might resonate within a phenomenological description that demands a phenomenological nod affirming recognition of the interpretation (van Manen, 1997).
